# A cell pattern approach to interpretation of fine needle aspiration cytology of thyroid lesions: A cyto-histomorphological study

**DOI:** 10.4103/0970-9371.73295

**Published:** 2010-10

**Authors:** Basavaraj P Bommanahalli, Ramachandra V Bhat, R Rupanarayan

**Affiliations:** Department of Pathology, SS Institute of Medical Sciences and Research Centre, Davangere – 577 005, Karnataka, India; 1 Department of Pathology, Indira Gandhi Medical College, Puduchery, India; 2 Department of Pathology, Sri Devaraj Urs Medical College, Kolar, Karnataka, India

**Keywords:** Cell pattern, fine needle aspiration cytology, thyroid

## Abstract

**Aim:**

Our study aimed at a cell pattern approach to interpret thyroid cytology and to demonstrate diagnostic accuracy of fine needle aspiration cytology (FNAC) with an emphasis on diagnostic pitfalls.

**Materials and Methods:**

A total number of 218 goitre cases, from the year 2000 to 2004, were reviewed retrospectively from the cytology files, without considering the previous cytological diagnosis. Four cases with inadequate aspirate were excluded. The predominant cell pattern, such as macro/normofollicular, microfollicular, papillary, syncytial, dispersed and cystic pattern, was noted in each case. The final diagnosis was arrived by observing the cellular details and background elements. Cytological diagnosis was correlated with histopathology in 75 cases. The sensitivity and specificity were computed.

**Results:**

Normo/macrofollicular pattern was seen in 71.96% of nodular goitre and 6.9% of follicular neoplasms. Around 86.2% of follicular neoplasms and 17.6% of papillary carcinoma had microfollicular pattern. The papillary pattern was seen in 47% of papillary carcinoma. Syncytial pattern was noticed in 72.3% of chronic lymphocytic thyroiditis and 29.4% of papillary carcinoma. Cytological diagnosis was concordant with histopathological diagnosis in 65 cases. Overall sensitivity and specificity of FNAC in diagnosing neoplastic lesions of thyroid were 83.33 and 95.55%, respectively.

**Conclusion:**

FNAC is more sensitive and specific in triaging neoplastic from non-neoplastic thyroid lesions. Identification of the predominant cell pattern would be complementary to analysis of cell morphology and background details in cytological diagnosis of thyroid lesions. This approach helps to diagnose follicular neoplasm and follicular variant of papillary thyroid carcinoma.

## Introduction

Goitre is a common clinical presentation with a prevalence rate of 4–7% in the general population.[1] Fine needle aspiration cytology (FNAC) is now a well-established, first-line, simple and quick screening test as well as the diagnostic tool for triaging surgical and non-surgical goitres.[2–4] Limitation of FNAC is mainly because of inadequate sampling, inexperience of the pathologist and overlapping cytological features mainly in samples obtained from hyperplastic nodule and follicular neoplasm.[5,6] In the aspirate, follicular epithelial cells might be arranged in many patterns like normo/macrofollicular, microfollicular, papillary, syncytial, dispersed cell and cystic pattern, depending upon the type of lesion. Therefore, in our study, the cytological diagnosis was approached, looking into the predominant type of cell pattern and observing cellular details and the background elements. Predominant cell pattern and cytological diagnosis were correlated with the histopathological diagnosis, and diagnostic pitfalls of FNAC have been discussed.

## Materials and Methods

We conducted a retrospective study in the department of pathology. A total of 218 goitre cases, from the year 2000 to 2004, were reviewed from the cytology files. On an average, in each case, there were two to three hematoxylin and eosin (H and E) stained slides, one to two giemsa and one to two Papanicolaou stained slides. A single pathologist reviewed all the slides and he was blinded about the previous cytological diagnosis in order to have unbiased diagnosis. Later, his diagnosis was compared with previous cytological diagnosis. Four cases with inadequate aspirate even after repeat FNAC were excluded. Spectrum of predominant cell pattern (macro/normofollicular, microfollicular, papillary, syncytial, dispersed and cystic pattern) was noted in each case. The final diagnosis was arrived by observing the cellular details and background elements.

Among these 214 cases, histopathology of 75 cases was available. The histopathological diagnosis was correlated with the cytological diagnosis and predominant cell pattern in FNAC. The sensitivity and specificity of cytological diagnosis in triaging neoplastic and non-neoplastic thyroid lesions were computed. Reasons for false positive and false negative results were evaluated and discussed.

## Results

Predominant cell patterns in 214 cases were as follows [Table 1].

**Table 1:** Distribution of predominant cell patterns in different thyroid lesions

Cytology diagnosis	Predominant cellular pattern						
	Normo/macrofollicular	Microfollicular	Papillary	Syncytial	Cystic	Dispersed	Total
Nodular goitre	77 (71.96)	02 (1.87)	–	–	28 (26.17)	–	107
Colloid cyst	–	–	–	–	11 (100)	–	11
Thyroiditis	–	06 (12.7)	–	34 (72.3)	–	07 (15)	47
Thyroglossal cyst	–	–	–	–	01 (100)	–	1
Follicular neoplasm	02 (6.9)	25 (86.2)	01 (3.45)	–	01 (3.45)	–	29
Papillary Ca	–	03 (17.6)	08 (47)	05 (29.4)	01 (6)	–	17
Anaplastic Ca	–	–	–	01 (100)	–	–	1
Medullary Ca	–	–	–	01 (100)	–	–	1

Total	79	36	09	41	42	07	214

Figures in parentheses indicates percentage; Ca-carcinoma

### The normo/macrofollicular pattern

**Figure 1:**
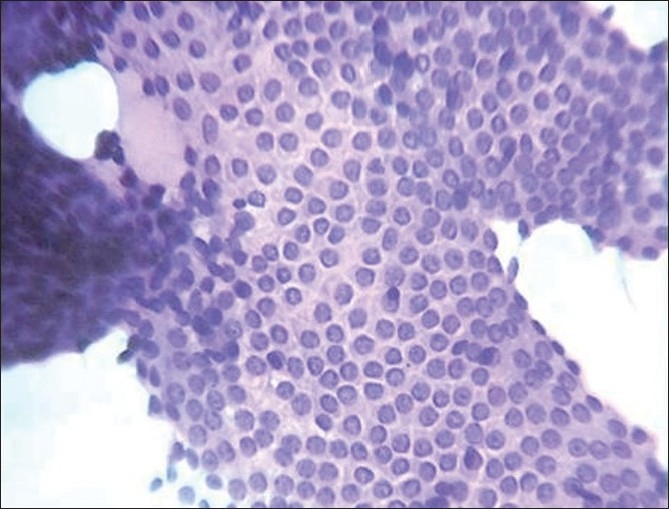
Follicular cells in normo/macrofollicular pattern (H and E, ×400)

The normo/macrofollicular pattern is characterised by sheets, clusters, or intact follicles.[2,7] This pattern was seen in 71.96% of nodular goitre and 6.9% of follicular neoplasms [Figure 1].

### Microfollicular pattern

**Figure 2:**
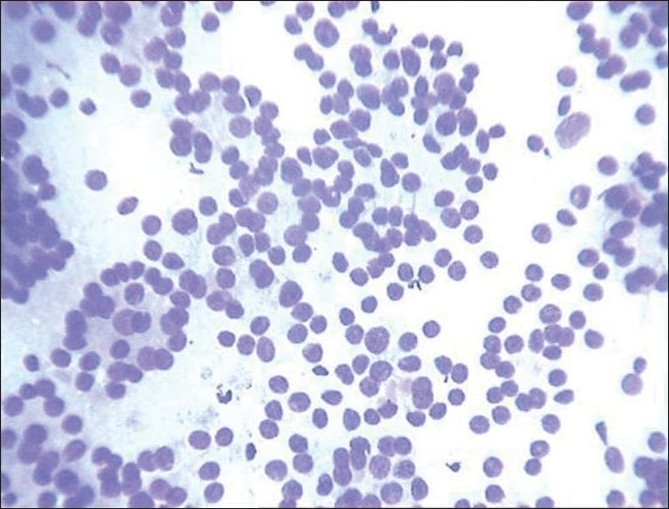
Follicular cells in microfollicular pattern (Giemsa, ×400)

A microacinar arrangement of follicular cells without well-defined lumen is referred to as rosette. When lumen is well defined, it is called as microfollicle [Figure 2]. Rosette and microfollicles represent the cross-sectional view of small follicles. Longitudinal view is referred to as tubule.[2,6,7] Around 86.2% of follicular neoplasms, 17.6% of papillary carcinoma and 12.7% of chronic lymphocytic thyroiditis had microfollicular pattern.

### Papillary pattern

**Figure 3:**
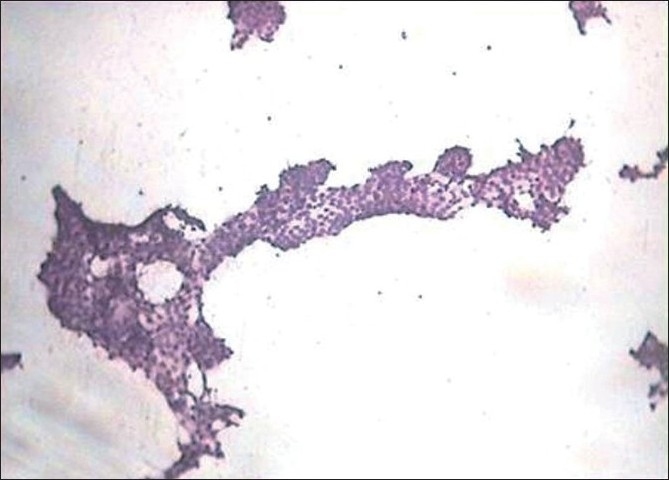
Follicular cells in papillary pattern (H and E, ×100)

Fibrovascular stalk with single or multilayered epithelial cells showing peripheral palisading of nuclei is called as papilla [Figure 3]. Papillae may show branching. Some of them are abortive without fibrovascular core.[2,7] The papillary pattern was seen in 47% of papillary carcinoma and 3.45% of follicular neoplasms.

### Syncytial pattern

**Figure 4:**
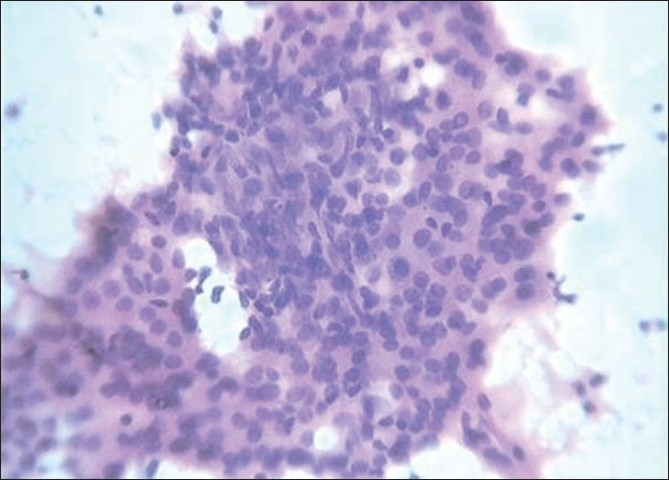
Follicular cells, Hürthle cells and lymphocytes arranged in syncytial pattern (H and E, ×400)

Sheets of follicular cells with loss of polarity and lack of distinct cell borders is called syncytial pattern [Figure 4].[7] This pattern was noticed in 72.3% of chronic lymphocytic thyroiditis, 29.4% of papillary carcinoma and all the cases of anaplastic carcinoma and medullary carcinoma.

### Dispersed cell pattern

**Figure 5:**
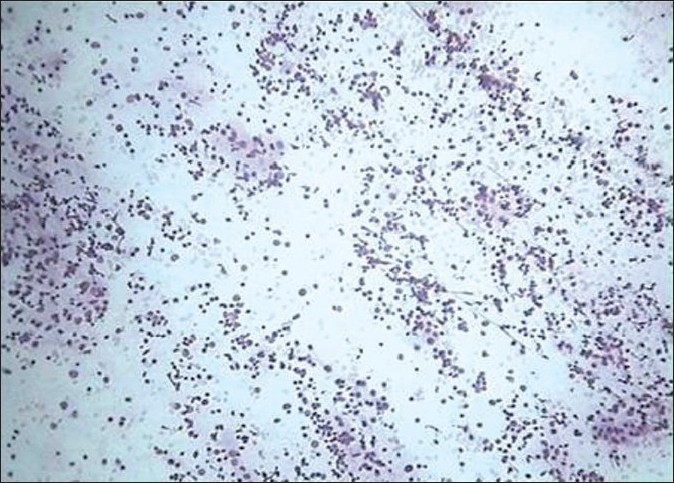
Follicular cells and lymphocytes are in dispersed cell pattern (H and E, ×100)

Loosely cohesive clusters and follicular cells in singles are referred to as the dispersed cell pattern [Figure 5].[7] It was observed in 15% of chronic lymphocytic thyroiditis.

### Cystic pattern

**Figure 6:**
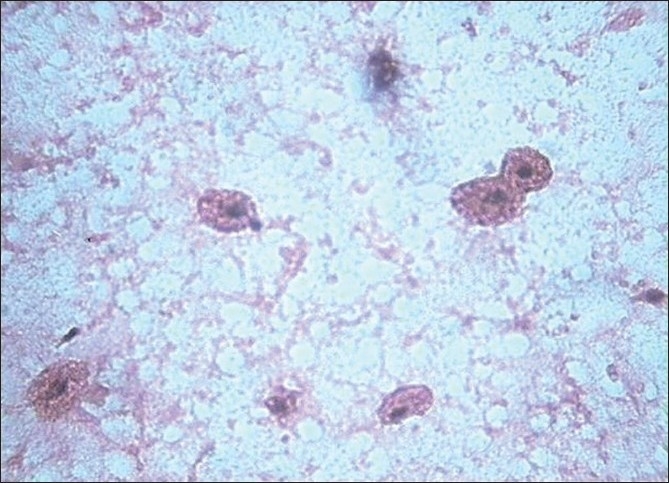
Hemosiderin macrophages in granular background – cystic pattern (H and E, ×400)

The cystic pattern in the thyroid aspirate is characterised by presence of hemosiderin laden macrophages in a granular background [Figure 6].[2,7] In our study, the cystic pattern was seen in 26.17% of nodular goitre, 6% of papillary carcinoma, all cases of colloid cyst and in a case of thyroglossal cyst.

Among the 214 cases, surgical specimens of 75 cases were available. The cyto-histopathological correlation [Table 2] was done. Concordance of diagnosis was seen in 37 cases of nodular goitre. Twenty-one of 37 cases had predominance of normo/macrofollicular pattern and 16 cases had cystic pattern in cytology. Discordance was seen in four cases; among them, two cases were confirmed as papillary carcinoma and other two as follicular adenoma – macrofollicular type.

**Table 2:** Cyto-histomorphological correlation

Cell pattern in cytology	Cytological diagnosis	Histopathological diagnosis	
		Concordance	Discordance
Normo/macrofollicular	24: Nodular goitre	21	1: Papillary Ca with MNG
			2: Follicular adenoma
Microfollicular	17: Follicular neoplasm	12	3: Papillary Ca
			2: Hyperplastic nodule MNG
	1: Nodular goitre	–	1: Follicular adenoma
	1: Thyroiditis	1	–
	2: Papillary Ca	2	–
Syncytial	4: Thyroiditis	4	–
	2: Papillary Ca	2	–
	1: Medullary Ca	1	–
Papillary	4: Papillary Ca	4	–
Cystic	17: Nodular goitre	16	1: Papillary Ca
	1: Papillary Ca	1	–
Dispersed	1: Thyroiditis	1	–

Total	75	65	10

Ca = Carcinoma, MNG = Multinodular goitre

All the six cases of thyroiditis were cyto-histopathologically concordant. Among them, syncytial pattern (4/6) was common. Twelve cases of cytologically diagnosed follicular neoplasms were confirmed by histopathology; among them two were follicular carcinoma. The predominant cell pattern observed in cytology was microfollicular architecture. Discordance with histopathology was seen in five cases of cytologically diagnosed follicular neoplasms. Out of them, three were confirmed as follicular variant of papillary thyroid carcinoma (FVPTC) and two as hyperplastic nodule in nodular goitre. All cytologically diagnosed papillary carcinomas were concordant with histopathology diagnosis. Among them, four showed papillary pattern.

Overall sensitivity and specificity of FNAC in diagnosing neoplastic lesions of thyroid were 83.33 and 95.55%, respectively. The positive predictive value and negative predictive value in diagnosing neoplasms were 92.59 and 89.58%, respectively.

### Comparison of the reviewer’s cytodiagnosis with the original cytodiagnosis

The original cytological diagnosis recorded in the cytology files was correlating with reviewer’s diagnosis in 205 cases, which included 104 nodular goitre, 11 colloid cysts, 47 thyroiditis, 26 follicular neoplasms, 14 papillary carcinomas, 1 anaplastic carcinoma, 1 medullary carcinoma and 1 thyroglossal cyst. Among the nine discordant cases, the original cytological diagnosis was follicular neoplasm in six cases and hyperplastic nodules in three cases. The reviewer’s diagnosis in these nine cases was three FVPTC, three hyperplastic nodules and three follicular neoplasms. Histopathology of three of nine discordant cases was available. Out of them, one case was hyperplastic nodule and two cases were FVPTC. These findings were concordant with reviewer’s diagnosis. In these three cases, the original diagnosis was follicular neoplasm.

## Discussion

Thyroid enlargement, whether diffuse or in the form of nodule, has to be investigated to rule out neoplasm. This definitely avoids unnecessary surgeries and reduces unnecessary burden on the healthcare system. FNAC is the first line of investigation along with other investigations like radioiodine scan, ultrasonography, hormonal assay and antibody levels.[8] However, most studies have reported high accuracy rates of FNAC in the diagnosis of neoplasm and thyroiditis.[3,9]

Presence of five to six groups of well-preserved follicular cells, with each group containing 10 or more cells on at least two slides from different passes satisfies the adequacy of the aspirate.[10] Fine needle capillary sampling of goitre may yield good cellularity.[11] In our study, four cases could not be reported because of inadequate sampling. The low frequency of “unsatisfactory for diagnosis” would result from a combination of a good aspiration technique and immediate examination of specimen adequacy by the cytopathologist.[12] The key to interpretation of thyroid FNAC is largely dependent on recognition of various cellular patterns, details of follicular cells and background elements like colloid and cyst macrophages.

The differential diagnoses of smears with predominantly normo/macrofollicular pattern most often include nodular goitre and follicular neoplasm with normo/macrofollicular pattern.[2,7] The presence of degenerative changes in monolayered sheets and abundant colloid would suggest possibility of non-neoplastic lesion.[13] In our study, normo/macrofollicular pattern was predominant (21/24) in nodular goitre. One of three discordant cases was histopathologically confirmed as papillary carcinoma with multinodular goitre. The reason for misdiagnosis could be a geographical miss when FNAC was done. Mathur *et al*.[14] found similar sampling errors responsible for missed diagnosis of papillary carcinoma. This geographical miss could be resolved by ultrasound guided aspiration.[2,12]

Two of three discordant cases in our study were follicular adenomas. This discordance was due to the presence of normo/macrofollicles and abundant colloid. Similar pitfall in diagnosis was encountered by Clary *et al*.[6] The necrotic material in the aspirate might be misinterpreted as thick colloid.[14]

The presence of predominant microfollicular pattern in smears could be seen in hyperplastic nodule, follicular neoplasm, FVPTC and thyroiditis.[6,7] These lesions are called as follicular lesions. In our study, microfollicular pattern was predominantly seen in follicular neoplasm. Greaves *et al*.[15] reviewed 96 FNAC cases of “follicular lesion” and reported a 30% malignancy rate, whereas 23% malignancy rate was reported by Ersoz *et al*.[16] who examined 56 cases of “solitary cellular nodules” and “cellular microfollicular lesions”. In our study, an 88.2% neoplastic rate was reported with 29.4% malignancy rate in follicular lesions. Cellularity, nuclear size, pleomorphism of cells and amount of colloid are helpful to distinguish neoplastic from non-neoplastic follicular lesions. Absence of colloid strongly favors diagnosis of neoplasm.[17] In follicular neoplasm, nuclear enlargement, if present, is generally a uniform change involving most or all of the cells from the lesion. Cells from hyperplastic nodular goitre and Hashimoto’s thyroiditis may show nuclear enlargement and pleomorphism, but this is usually a focal phenomenon. Prominent nucleoli, necrotic debris in the background, if present, are highly suggestive of carcinoma.[18,19] Two of our cases showed pleomorphism with prominent nucleolus and were histopathologically confirmed as follicular carcinoma. Sub-classification of variants in papillary thyroid carcinoma is possible based on the cellular architecture.[20] Papillary structures are rarely seen in FVPTC. However, Leung *et al*.[21] and Das *et al*.[2] observed papillary structures in 53.8 and 50%, respectively, of FVPTC. In our present study, this feature was seen in two cases. Williams *et al*.[22] suggested that nuclear features like nuclear grooves, intranuclear cytoplasmic inclusions and powdery chromatin were statistically significant in the majority of FVPTC. But these features were not retained in some of the smears; this difference is possibly related to fixation techniques.[23] Diagnosis of FVPTC could be missed in the absence of characteristic nuclear features of PTC even though presence of high cellularity, microfollicular pattern and less colloid favors diagnosis of follicular neoplasm.[20,23,24] In our study, we diagnosed two cases of FVPTC but missed diagnosis in three cases because of the absence of nuclear features.

Follicular cell in syncytial pattern could be seen predominantly in neoplastic conditions like PTC, follicular/Hürthle cell neoplasm and medullary carcinoma, along with thyroiditis.[7] In our study, this pattern was seen in thyroiditis, PTC and medullary carcinoma. Observations of “lymphocytic hug onto the follicular cells” and cellular details are useful to differentiate between the differential diagnoses.

The presence of papillae and pseudopapillae in aspirate hint differential diagnoses of Graves’ disease, hyperplastic nodule and papillary carcinoma.[7] Characteristic nuclear features like transpolar nuclear grooves in more than or equal to 20% of follicular cells is virtually diagnostic of neoplasm, most likely papillary carcinoma.[20,25] Even the presence of more than three intranuclear inclusions in the enlarged nuclei on single aspirate is almost pathognomonic of PTC.[26] Therefore, nuclear features and high cellularity are useful in diagnosing papillary carcinoma.[24] Cystic change could be seen in developmental/congenital, hyperplastic and neoplastic entities like cystic PTC and rarely in follicular neoplasm. Only 10–15% of the cysts are neoplastic; however, papillary thyroid carcinomas are important because of false negative diagnosis in FNAC.[7] After aspiration of fluid, every attempt should be made to obtain aspirate from solid area. Here, guided FNAC is more useful to aspirate from solid lesion.[2,12] In our study, incidence of cystic neoplasms was 12.5% (2/16). Misinterpretation of cystic papillary carcinoma in cytology was mainly because of less cellularity and absence of characteristic nuclear features.

Differential diagnoses for follicular cells in dispersed cell pattern could be thyroiditis and neoplastic lesions like hyalinizing trabecular adenoma, Hürthle cell neoplasms, medullary carcinoma and lymphomas.[7] This pattern was seen in thyroiditis in the present study.

According to our observation, syncytial pattern, papillary pattern and microfollicular pattern were more common in neoplasms and thyroiditis. The predominant cell pattern raises differential diagnoses in the mind of pathologist, which could be easily narrowed to final diagnosis according to the cyto-morphological features and background elements like colloid or cyst macrophages. Noting the cell pattern would help to identify variants of PTC, follicular neoplasm and other thyroid lesions. Therefore, identification of the predominant cell pattern would be complementary to analysis of cell morphology and background details in the cytological diagnosis of thyroid lesions. Grey zones of diagnostic difficulty still exist especially in follicular lesions because of overlap of cell pattern, cellular details and background elements.

## Conclusions

FNAC of thyroid is cost-effective, simple and out-patient based investigation. It is more sensitive and specific in triaging neoplastic from non-neoplastic thyroid lesions. Adequacy of aspirate is the first step toward making the correct diagnosis. Observing the cell pattern at the outset is an easy and convenient and complementary way of thyroid cytological study before analyzing cellular details and background elements. Syncytial pattern, papillary pattern and microfollicular pattern are more commonly seen in neoplasms and thyroiditis. Cell pattern approach helps to identify thyroid lesions, follicular neoplasms and variants of PTC, especially FVPTC. This approach could be a very good step for a beginner in cytology and also for the departments with volumes of FNAC cases to screen the slides. Pitfalls in thyroid cytology could be resolved by correct sampling from lesion with meticulous examination.

## References

[1] Rojeski MT, Gharib H (1985). Nodular thyroid disease: Evaluation and management. N Engl J Med.

[2] Das KD, Khanna CM, Tripathi RP, Pant CS, Mandal AK, Chandra S (1999). Solitary nodular goiter: Review of cytomorphologic features in 441cases. Acta Cytol.

[3] Afroze N, Kayani N, Hassan SH (2002). Role of fine needle aspiration cytology in diagnosis of palpable thyroid lesions. Indian J Pathol Microbiol.

[4] Handa U, Garg S, Mohan H, Nagarkar N (2008). Role of fine needle aspiration cytology in diagnosis and management of thyroid lesions: A study on 434 patients. J Cytol.

[5] Jagoi S, Al- Jassar A, Temmim L, Dey P, Adesina AO, Amanguno HG (2005). Fine needle aspiration cytology of the thyroid: a cytohistological study with evaluation of discordant cases. Acta Cytol.

[6] Clary KM, Condel JL, Liu Y, Johnson DR, Grzybicki DM, Raab SS (2005). Interobserver variability in the fine needle aspiration biopsy diagnosis of follicular lesions of the thyroid gland. Acta Cytol.

[7] Nayar R, Frost AR (2001). Thyroid aspiration cytology: a “cell pattern” approach to interpretation. Semin Diagn Pathol.

[8] Hegedus L (2004). Clinical practice. The thyroid nodule. N Eng J Med.

[9] Ko HM, Jhu IK, Yang SH, Lee JH, Nam JH, Juhng SW (2003). Clinicopathologic analysis of fine needle aspiration cytology of the thyroid. A review of 1,613 cases and correlation with histopathologic diagnoses. Acta Cytol.

[10] Hamburger JI, Hussain M (1988). Semiquantitative criteria for fine needle biopsy diagnosis: reduced false negative diagnoses. Diagn Cytopathol.

[11] Kamal MM, Arjune DG, Kulkarni HR (2002). Comparative study of fine-needle aspiration and fine needle capillary sampling of thyroid lesions. Acta Cytol.

[12] Solymosi T, Toth GL, Bodo M (2001). Diagnostic accuracy of fine needle aspiration cytology of thyroid: impact of ultra-sonography and ultrasonographically guided aspiration. Acta Cytol.

[13] Suen KC (1988). How does one separate cellular follicular lesions of the thyroid by fine-needle aspiration biopsy?. Diagn Cytopathol.

[14] Mathur SR, Kapila K, Verma K (2005). Role of fine needle aspiration cytology in the diagnosis of goiter. Indian J Pathol Microbiol.

[15] Greaves TS, Olvera M, Florentine BD, Raza AS, Cobb CJ, Tsao-Wei DD (2000). Follicular lesions of thyroid: a-5-year fine-needle aspirate experience. Cancer.

[16] Ersoz C, Firat P, Uguz A, Kuzey GM (2004). Fine-needle aspiration cytology of solitary thyroid nodules: how far can we go in rendering differential diagnoses?. Cancer.

[17] Somma J, Schlecht NF, Fink D, Khader SN, Smith RV, Cajigas A (2010). Thyroid fine needle aspiration cytology: follicular lesions and the gray zone. Acta Cytol.

[18] Goldstein RE, Netterville JL, Burkey B, Johnson JE (2002). Implications of follicular neoplasms, atypia and lesions suspicious for malignancy diagnosed by fine-needle aspiration of thyroid nodules. Ann Surg.

[19] Kaur A, Jayaram G (1991). Thyroid tumors: cytomorphology of follicular neoplasms. Diagn Cytopathol.

[20] Gupta S, Sodhani P, Jain S, Kumar N (2004). Morphologic spectrum of papillary carcinoma of the thyroid: role of cytology in identifying the variants. Acta Cytol.

[21] Leung CS, Hartwick RW, Bédard YC (1993). Correlation of cytologic and histologic features in variants of papillary carcinoma of the thyroid. Acta Cytol.

[22] Goodell WM, Saboorian MH, Ashfaq R (1998). Fine-needle aspiration diagnosis of follicular variant of papillary carcinoma. Cancer.

[23] Shih SR, Shun CT, Su DH, Hsiao YL, Chang TC (2005). Follicular variant of papillary carcinoma: diagnostic limitations of fine needle aspiration cytology. Acta Cytol.

[24] Das DK, Sharma PN (2009). Diagnosis of papillary thyroid carcinoma in fine needle aspiration smears: factors that affect decision making. Acta Cytol.

[25] Yi JY, Sukru SD (2003). Evaluating diagnostic significance of nuclear grooves in thyroid fine needle aspirates with a semi-quantitative approach. Acta Cytol.

[26] Sanchez AM, Stahl ER, Koss LG, Melamed MR (2006). Thyroid, parathyroid and neck masses other than lymph nodes. Koss’s diagnostic cytopathology and its histopathologic bases.

